# Exosomal miR-150 partially attenuated acute lung injury by mediating microvascular endothelial cells and MAPK pathway

**DOI:** 10.1042/BSR20203363

**Published:** 2021-12-23

**Authors:** Jiaxin Xu, Dan Xu, Zhizhong Yu, Zhaohui Fu, Zheng Lv, Lei Meng, Xin Zhao

**Affiliations:** Department of Critical Care Medicine, Union Hospital, Tongji Medical College, Huazhong University of Science and Technology, Wuhan 430022, China

**Keywords:** acute lung injury, CD34, exosomes, miR-150, VE-cadherin

## Abstract

Background: Acute lung injury (ALI) is a respiratory disease with high morbidity and mortality rates. Currently, there is no effective treatment to complement mechanical ventilation. Exosomes and microRNAs (miRNAs) are promising agents for the management of this disease.

Methods: Exosomes were isolated from mouse bone marrow stromal stem cells (BMSCs). The levels of two miRNAs, miR-542-3P and miR-150, in exosomes were determined using RT-PCR, and miR-150 was selected for further study. ALI model was established in mice using lipopolysaccharides, and then, they were treated with saline, exosomes, miRNA agomirs, or miRNA antagomirs. The concentrations of TNF-α, IL-6, and IL-1β and the number of neutrophils and macrophages in the bronchoalveolar lavage fluid were measured. The wet/dry weight ratio of the lung tissue was calculated, and tissue pathology and apoptosis were observed using hematoxylin and eosin and terminal deoxynucleotidyl transferase dUTP nick-end labeling staining. CD34 and VE-cadherin expression was detected using immunofluorescence. Proteins associated with apoptosis and MAPK signaling were detected using Western blotting, and miR-150 expression in lung tissue was evaluated using RT-PCR.

Results: We successfully isolated BMSCs and exosomes and showed that the level of miR-150 was significantly higher than that of miR-542-3p. Exosomes and miR-150 reduced inflammation and lung edema while maintaining the integrity of the alveolar structure. They also mitigated microvascular endothelial cell injury by regulating the caspase-3, Bax/Bcl-2, and MAPK signaling.

Conclusions: Exosomal miR-150 attenuates lipopolysaccharide-induced ALI through the MAPK pathway.

## Introduction

Mesenchymal stem cells (MSCs) are derived from the mesoderm and are capable of self-renewal and multi-differentiation. They exist in multiple tissues, including the bone marrow, amniotic fluid, umbilical cord blood, and placenta [1]. MSCs are highly proliferative, are stably passaged, and possess low immunogenicity, anti-inflammatory properties, and homing ability. They efficiently express transfected genes, regulate immune responses, and secrete nutrients, thus exhibiting advantageous prospects for the treatment of various diseases [2]. At present, MSCs have been applied in the treatment of a variety of clinical diseases, including acute myocardial infarction [3], liver fibrosis [4], gastric cancer [5], breast cancer [6], lung cancer [7], and systemic lupus erythematosus [8].

Among MSCs, bone marrow-derived mesenchymal stem cells (BMSCs) are one of the most common types that possesses a number of advantages [9–11]. However, when BMSCs are delivered into the body, their proliferative capacity is weakened, which in turn lowers cell number, posing a risk of carcinogenesis and limiting their application [12]. In recent years, an increasing number of studies have demonstrated that BMSCs exert their functions through paracrine mechanisms [13] and that BMSC-conditioned media have anti-inflammatory and anti-apoptotic functions that could aid damage repair [14]. Microvesicles were later found in BMSC-conditioned media, and exosomes were identified among them. Exosomes are double-membrane carriers that contain a variety of functional factors, such as microRNAs (miRNAs) and proteins. Studies have reported that exosomes carry miRNAs to injured tissues to reduce damage [15]. These molecules are small noncoding RNAs that silence targeted genes by base pairing to partially or totally complementary sequences in the 3′-untranslated regions of the target mRNA [16].

Patients with acute lung injury (ALI) and acute respiratory distress syndrome (ARDS) experience epithelial and endothelial cell damage and increased vascular permeability [17]. Previous studies have reported that miRNA-542-3p could suppress tumor angiogenesis by targeting angiopoietin, and miRNA-150 ameliorated vascular injury by increasing the generation of Ang-2 [18,19]. In addition, serum miR-150 expression is a potential biomarker for diagnosis and prognosis of ALI, and miR-150 has been found to suppress lipopolysaccharide (LPS)-induced ALI by regulating AKT3 expression [20,21]. Our study investigated the roles of these two miRNAs in the treatment of ALI with BMSC-derived exosomes.

## Materials and methods

### BMSC culture

All animal experiments were conducted in accordance with the National Institutes of Health Guide for the Care and Use of Laboratory Animals (NIH Publications No. 8023, revised 1978). All experimental procedures were performed at Huazhong University of Science and Technology Animal House and approved by the Animal Ethics Committee of Huazhong University of Science and Technology. C57Bl/6J mice (male, 6-week-old) were obtained from Beijing HFK Bioscience Co., Ltd. The mice were maintained at 24°C and 40% humidity and were provided free access to water and feed. BMSCs were isolated from these mice as previously described and cultured in low-glucose Dulbecco’s modified Eagle medium containing 10% fetal bovine serum, 1% penicillin–streptomycin, and 1% L-glutamine under the conditions of 5% CO_2_ at 37°C [22]. Non-adherent cells were discarded, and the growth medium was replaced every 3–4 days. Cells were observed under an inverted microscope and passaged when they reached 80–90% confluence. Cells at passage 3 were photographed, subjected to flow cytometric identification of surface antigens (CD45, CD90, and CD105) (Beckman Coulter Cytomics FC500 MCL), and induced to differentiate using osteogenic and adipogenic media.

### Exosome isolation and characterization and identification of miRNAs

Exosomes were isolated as previously described and observed under a transmission electron microscope (H-600 HITACHI, Japan) [23]. After dilution with phosphate-buffered saline (PBS), the protein concentration in 1 μl of exosomes was quantified using a bicinchoninic acid assay kit, following the manufacturer’s instructions (Beyotime Institute of Biotechnology). The expression of miR-150 and miR-542-3p in the exosomes was measured using RT-PCR with the following primers: miR-150 forward 5′-GGGTCTCCCAACCCTTG-3′ and reverse 5′-AACTGGTGTCGTGGAGTCGGC-3′; miR-542-3p forward 5′-GGGTGTGACAGATTGAT-3′ and reverse 5′-AACTGGTGTCGTGGAGTCGGC-3′; and normalized to U6 snRNA forward 5′-CTCGCTTCGGCAGCACATATACT-3′ and reverse 5′-ACGCTTCACGAATTTGCGTGTC-3′.

### Mouse model of LPS-induced ALI

Mice were maintained under sterile conditions at 24°C and 40% humidity with free access to water and food. LPS was administered intratracheally at 4 mg/kg to establish the ALI model. The mice (*n*=30) were randomly divided into five groups (*n* = 6 per group): control (CON; no treatment), LPS, LPS + exosomes (ALI plus tail vein injection of 100 μg of exosomes in 0.2 mL), LPS + miR-150 agomir (ALI plus tail vein injection of 80 mg/kg of miR-150 agomirs in 0.2 ml), and LPS + miR-150 antagomir (ALI plus tail vein injection of 80 mg/kg of miR-150 antagomirs in 0.2 ml). The mice were anesthetized with an intraperitoneal injection of 1% pentobarbital sodium (sigma merck) at 0.12 ml/10 g, and lung samples were collected for the next experiment. The miR-150 agomirs and antagomirs were purchased from Guangzhou RiboBio Co., Ltd. The mice were killed with an intraperitoneal injection of pentobarbital sodium at 100 mg/kg, and death was confirmed with no heartbeat and respiration.

### Neutrophil count and measurement of cytokine and protein levels in bronchoalveolar lavage fluid (BALF)

BALF samples were obtained from normal or ALI-induced mice 48 h after treatment with or without exosomes, miRNA agomirs, and miRNA antagomirs. The numbers of total cells, neutrophils, and macrophages were counted using the Diff-Quik method. The total concentration of proteins in the BALF was determined using a bicinchoninic acid assay kit (Beyotime, China). The concentrations of tumor necrosis factor (TNF)-α, interleukin (IL)-6, IL-1β, and IL-10 in the BALF were measured using an enzyme-linked immunosorbent assay kit (R&D Systems).

### Lung wet/dry weight ratio and hematoxylin and eosin (H&E) and terminal deoxynucleotidyl transferase dUTP nick-end labeling (TUNEL) staining

Lung samples were weighed before and after drying in an oven at 60°C, and the wet/dry weight ratio was calculated. Lung samples were harvested and excised from normal or ALI-induced mice 48 h after treatment with or without exosomes, miRNA agomirs, or miRNA antagomirs. Before obtaining lung tissues, the mice were anesthetized and killed using pentobarbital sodium (Sigma-Aldrich) at 100 mg/kg of body weight. Samples were fixed in 4% paraformaldehyde, embedded in paraffin, cut into 4-μm-thick sections, and subjected to H&E and TUNEL staining (*In Situ* Apoptosis Detection kit, Roche Applied Science), according to the manufacturer’s instructions.

### Western blotting

Western blotting was performed to measure the protein levels of cell apoptosis and MAPK pathway proteins in lung tissues, as previously described [24]. The primary antibodies against the following proteins were used: caspase-3 (1:500, ab1384, Abcam), cleaved caspase-3 (1:500, ab49822, Abcam), Bax (1:3000, ab32503, Abcam), Bcl-2 (1:500, ab692, Abcam), Erk (1:1000, #4694, CST), p-Erk (1:2000, #4370, CST), JNK (1:1000, #4668, CST), p-JNK (1:1,000; ab179461; Abcam), p38 (1;1000, #9212, CST), p-p38 (1:1000, #9211, CST), and β-actin (1:1000, ab8226, Abcam). The following secondary antibodies were used: horseradish peroxidase-conjugated goat anti-mouse IgG H&L (1:2000, ab97023, Abcam) and goat anti-rabbit IgG H&L (1:2000, ab6721, Abcam).

### Immunofluorescence staining

Samples were fixed in 4% paraformaldehyde, embedded in paraffin, cut into 4-μm-thick sections, and incubated with blocking solution (3% bovine serum albumin and 0.2% Triton-X in PBS) at room temperature for 1 h. The cells were then incubated overnight at 4°C with primary antibodies against CD34 and vascular endothelial (VE)-cadherin diluted in PBS. Thereafter, secondary antibodies (Abcam) were added and incubated for 1 h at room temperature. The nuclei were stained with 4′,6-diamidino-2-phenylindole for 20 min at room temperature. Immunofluorescence was visualized using a fluorescence microscope (Nikon, Tokyo, Japan). The collected images were quantified using Image Pro-Plus 6.0 (Media Cybernetics, Silver Spring, MD, U.S.A.).

### Statistical analyses

All data are shown as the mean ± standard deviation. Data were analyzed using SPSS software, and figures were prepared using GraphPad Prism software. Comparisons between more than two groups were made using one-way analysis of variance with Duncan’s multiple comparisons test. Statistical significance was set at *P*<0.05.

## Results

### Characterization, isolation, and identification of BMSCs and BMSC-derived exosomes

**Figure 1:**
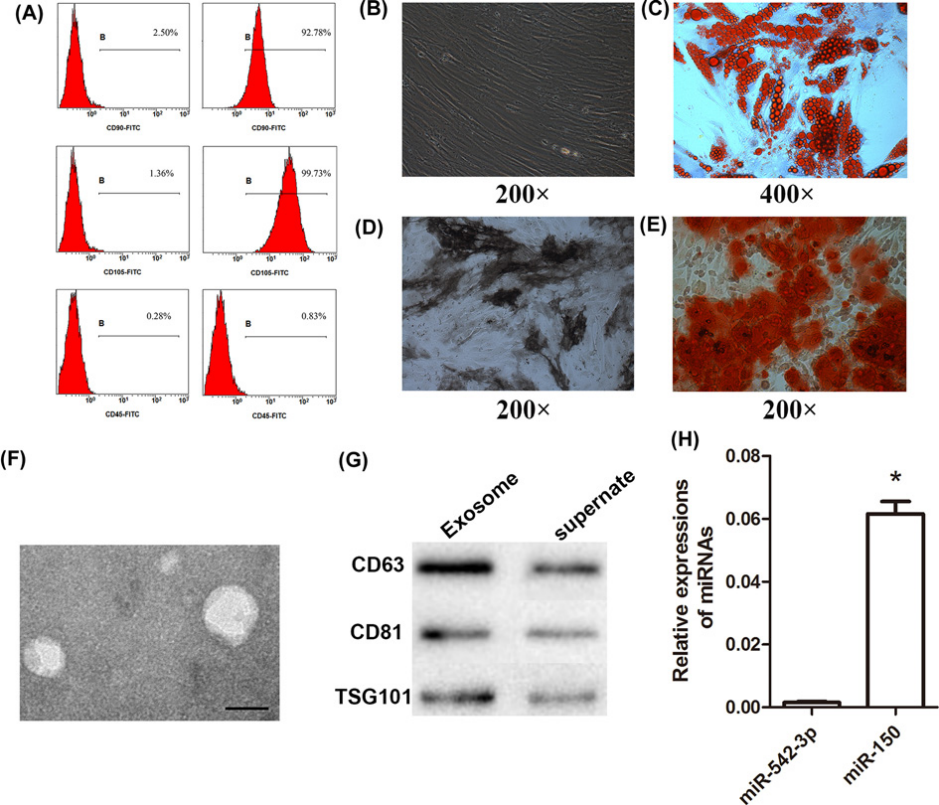
MSCs derived from the bone marrow of C57BL/6 mice and exosomes derived from BMSCs (**A**) Surface antigens (CD45, CD90, and CD105) of BMSCs were identified using flow cytometry. (**B**) BMSCs at passage 3 were observed under a light microscope. (**C**) BMSCs were differentiated using adipogenic media. (**D**, **E**) BMSCs were differentiated using osteogenic media. (**F**) Ultrastructural features of exosomes derived from BMSCs (scale bar: 60 nm). (**G**) Expression of exosome-specific proteins CD63, CD81, and TSG101. (**H**) Expression of miR-542-3p and miR-150 in exosomes derived from BMSCs; *P<0.05 vs. miR-542-3p.

The BMSCs were successfully isolated as demonstrated by the positive expression of CD90 and CD105, which are surface markers of BMSCs, but they did not express CD45 [25] (Figure 1A). The cells were well-cultured, and their cell morphology was in the form of long shuttles (Figure 1B). Then, we demonstrated the differentiation capability of BMSCs cultured in the special differentiation media. Our results showed that BMSCs successfully differentiated into fat cells and osteocytes (Figure 1C–E).

Exosomes secreted from BMSCs were isolated and photographed using an electron microscope. The exosomes were spherical and exhibited a double-membrane structure with a diameter of 30–100 nm (Figure 1F). Western blot assay showed that specific exosomal markers CD63, CD81, and TSG101 [26] were expressed in the exosomes, which further validated the successful isolation of exosomes (Figure 1G). Exosomes contain a variety of substances with specific functions, including miRNAs. As miR-542-3p and miR-150 are known to be involved in angiogenesis, we measured their expression in the isolated exosomes. The expression of miR-542-3p and miR-150 in exosomes was significantly different (Figure 1H); thus, we chose miR-150 for further studies.

### Exosomes and miR-150 inhibited LPS-induced pulmonary inflammation and permeability

**Figure 2:**
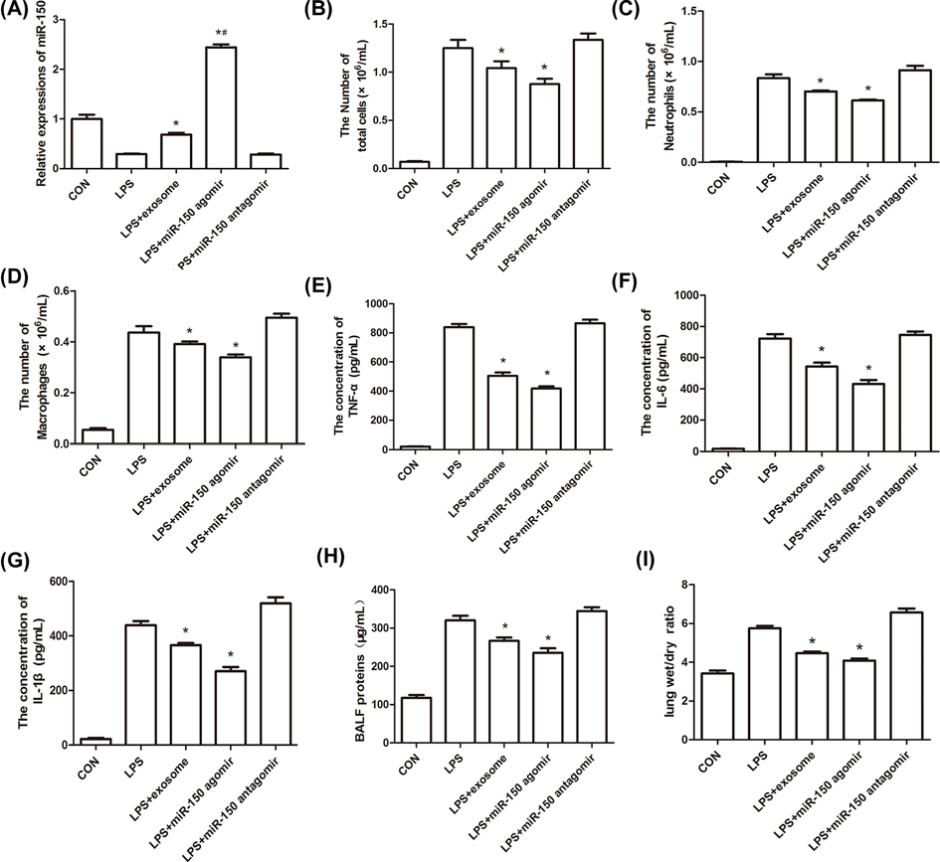
Effect of exosomes and miR-150 on inflammation and lung wet/dry weight ratio of LPS-induced ALI in mice (**A**) Expression of miR-150 in CON, LPS, LPS + exosome, LPS + miR-150 agomir, and LPS + miR-150 antagomir groups. The numbers of total cells (**B**), neutrophils (**C**), and macrophages (**D**) and the concentrations of TNF-α (**E**), IL-6 (**F**), IL-1β (**G**), and total proteins in the BALF (**H**) were measured. (**I**) Lung wet/dry weight were measured; *P<0.05 vs. LPS.

The miR-150 levels were estimated in different groups, and the results showed that LPS treatment inhibited the levels of miR-150, but exosomes increased its levels in BSMCs (Figure 2A). There was no significant difference between the CON group and the CON + exosome group (Supplementary Figure S1). The number of total cells, neutrophils, and macrophages, the concentrations of TNF-α, IL-6, IL-1β, and total proteins, and the wet/dry pulmonary weight were measured and are shown in Figure 2B–I. The results demonstrated that LPS treatment increased the number of total cells, neutrophils, and macrophages, the concentrations of TNF-α, IL-6, IL-1β, and total proteins, and the wet/dry pulmonary weight, which were decreased by exosomes and miR-150, indicating that exosomes and miR-150 significantly reduced pulmonary inflammation and permeability.

### Exosomes and miR-150 attenuated LPS-induced ALI and apoptosis

**Figure 3:**
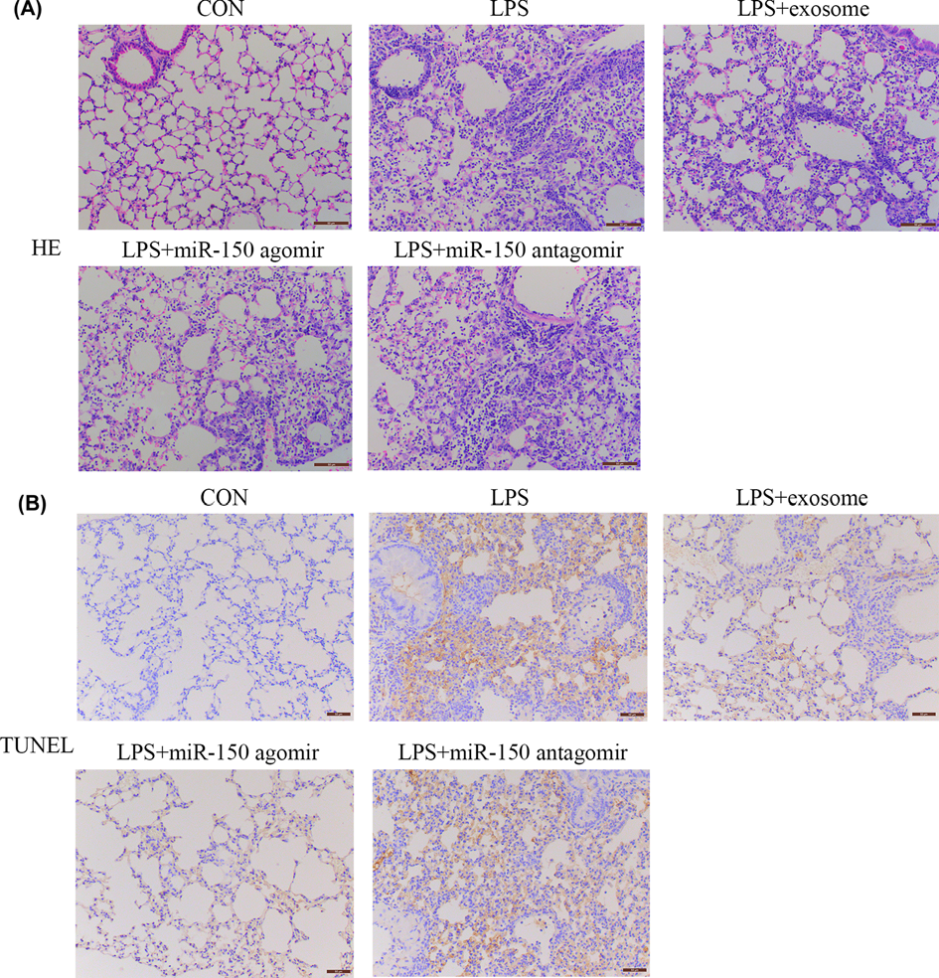
Effect of exosomes and miR-150 on histopathological changes and apoptosis in the lungs of mice Histological changes and apoptosis in the lungs of mice in the CON, LPS, LPS + exosome, LPS + miR-150 agomir, and LPS + miR-150 antagomir groups were observed using (**A**) H&E and (**B**) TUNEL, respectively (magnification 200×).

H&E staining was used to investigate changes in the lung structure (Figure 3A). In the CON group, the structure of the lung was clear, alveolar epithelial cells were intact, and there were no abnormalities, infiltration of inflammatory cells, or congestion. Compared with the CON group, LPS destroyed the alveolar structure of the lung and thickened the alveolar wall in the LPS-treated groups. After treatment with exosomes and miR-150, the alveolar structures recovered to some extent. TUNEL staining was used to detect apoptosis in the lungs (Figure 3B). We found that exosomes and miR-150 attenuated LPS-induced apoptosis. These results suggested that exosomes and miR-150 could repair injured lungs and inhibit cell apoptosis.

### Exosomes and miR-150 mitigated LPS-induced microvascular endothelial cell damage

**Figure 4:**
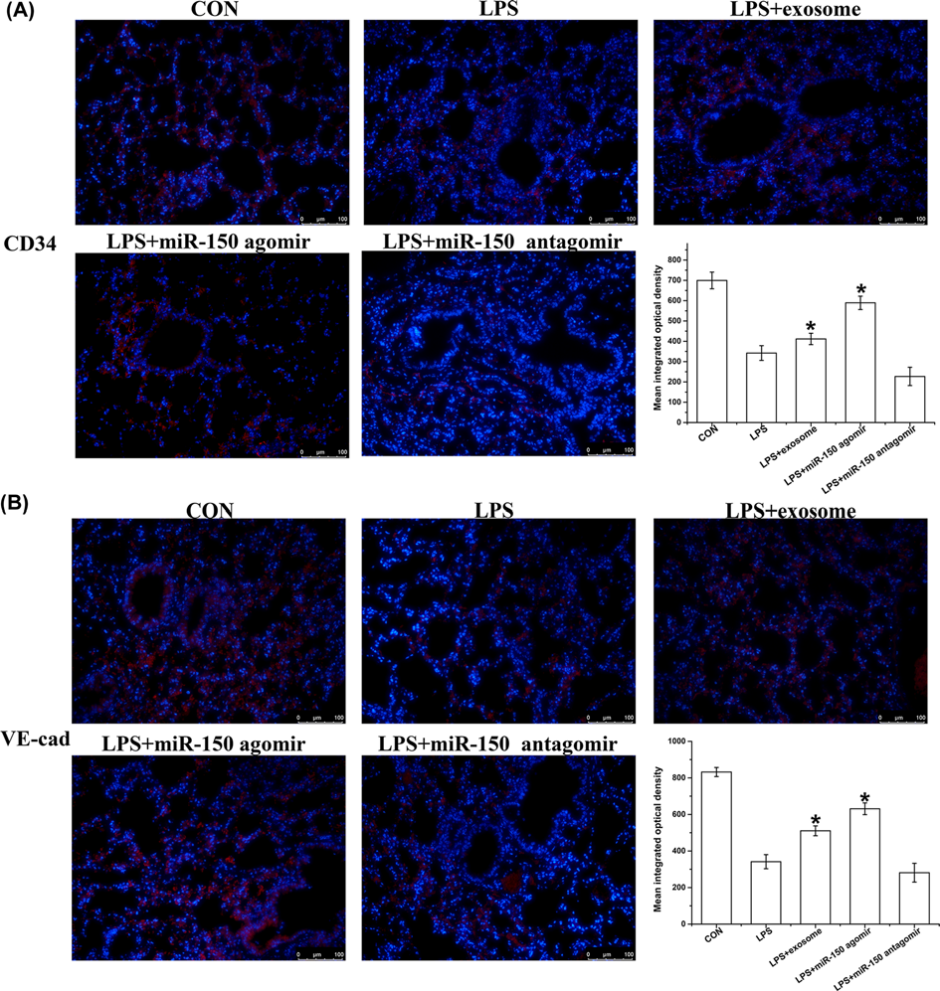
Effect of exosomes and miR-150 on the expression of CD34 and VE-cadherin in LPS-induced ALI in mice Representative images and quantification of (**A**) CD34 and (**B**) VE-cadherin expression obtained from mice in the CON, LPS, LPS + exosome, LPS + miR-150 agomir, and LPS + miR-150 antagomir groups, (magnification 200×), * *P*<0.05 vs. LPS.

Microvascular endothelial cells are key modulators of microvascular function, including the maintenance of microvascular permeability barriers. Thus, we observed two specific molecules of microvascular endothelial cells, CD34 and VE-cadherin. As shown in Figure 4, LPS caused a reduction in the expression of CD34 and VE-cadherin, whereas exosomes and miR-150 mitigated the LPS-induced loss of CD34 and VE-cadherin.

### Caspase-3, Bax/Bcl-2, and the MAPK pathway were modulated by exosomes and miR-150

**Figure 5:**
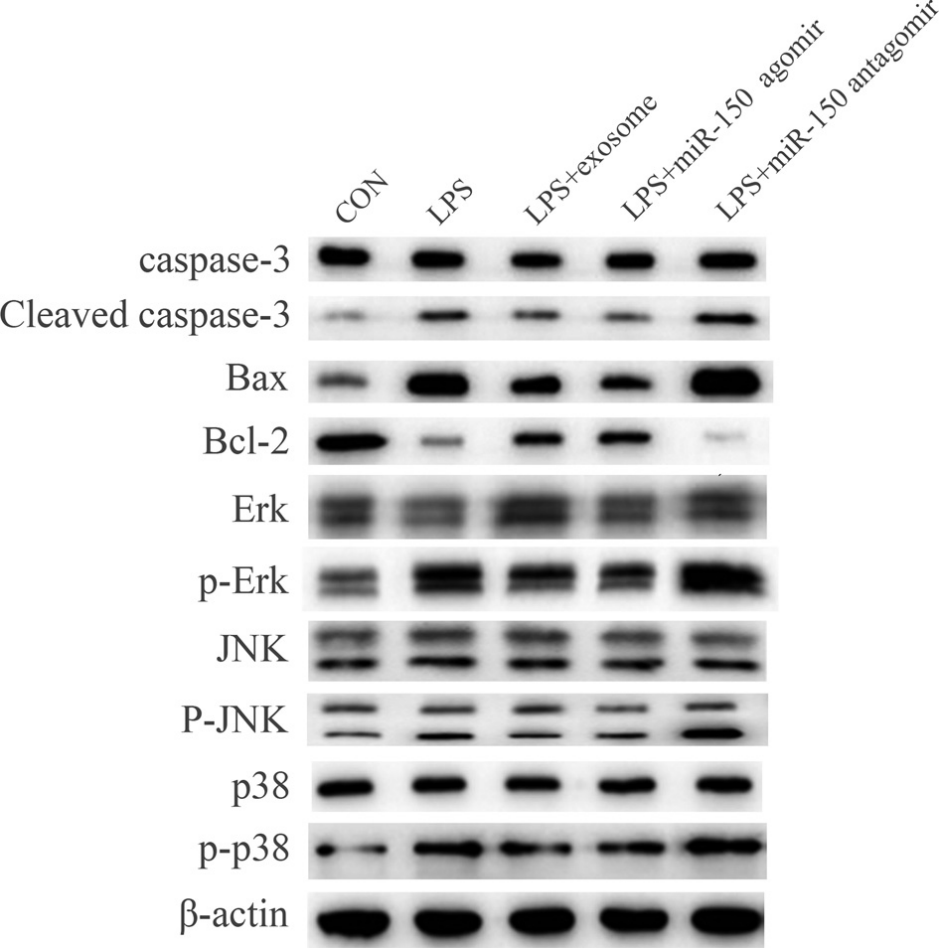
Effect of exosomes and miR-150 on the expression of caspase-3, Bax/Bcl-2, and MAPK-associated proteins in LPS-induced ALI in mice The protein expression levels of caspase-3, cleaved caspase-3, Bax, Bcl-2, Erk, p-Erk, JNK, p-JNK, p38, and p-p38 in the CON, LPS, LPS + exosomes, LPS + miR-150 agomir, and LPS + miR-150 antagomir groups were measured by Western blotting.

To understand the molecular mechanism underlying the repair functions of exosomes and miR-150, we measured the expression of caspase-3, Bax/Bcl-2, and MAPK-associated proteins (Figure 5). Compared with those in the control group, the expression of cleaved caspase-3, Bax, p-Erk, p-JNK, and p-p38 were increased in the LPS-treated group but were suppressed by exosomes and miR-150. The expression of Bcl-2 was inhibited by LPS; by contrast, exosomes and miR-150 increased the expression of Bcl-2.

## Discussion

BMSCs possess specific cell morphology and can be identified by specific cell surface antigens. Ma et al. [27] isolated MSCs from the bone marrow of Sprague–Dawley rats. These BMSCs were spindle-shaped and expressed the cell surface antigens CD29, CD44, and CD90 but not CD45. Qingqing et al. [28] showed that spindle-shaped BMSCs isolated from C57BL/6 mice showed negative expression of cell surface antigens CD34 and CD45 and positive expression of CD29, CD44, and CD105. In our study, the BMSCs were spindle-shaped, and the expression of cell surface antigens CD90 and CD105 was positive, whereas that of CD45 was negative, which was consistent with the above results.

When cultured *in vitro*, BMSCs can differentiate into adipocytes, osteoblasts, chondrocytes, nerve cells, and cardiomyocytes, which can be easily detected using oil red O, alkaline phosphatase, and alizarin red S staining. Gao et al. [29] isolated BMSCs from patients with age-related osteoporosis and induced their differentiation into adipocytes. After staining with oil red O, orange-red oil droplets were observed. The same results were observed in our study, demonstrating that BMSCs differentiated into adipocytes. Yang et al. [30] isolated BMSCs from Kunming mice. The BMSCs were stained with alkaline phosphatase (Gomori-modified calcium-cobalt method), and black cobalt sulfide was observed. BMSCs were also stained with alizarin red S, and red calcium nodules were observed. In our study, the same results were detected, showing that BMSCs differentiated into osteoblasts. The results shown in Figure 1 demonstrate that we successfully isolated BMSCs from C57BL/6 mice.

We further obtained exosomes from the isolated BMSCs and observed them using transmission electron microscopy. The exosomes showed a double-membrane structure with a diameter of 30–100 nm. As the expression of miR-150 in exosomes was significantly higher than that of miR-542-3p, we chose miR-150 for further studies.

In order to verify whether exosomes and miR-150 have beneficial effects in the management of LPS-induced ALI, we treated ALI mice with exosomes, miR-150 agomirs, and miR-150 antagomirs. Compared with the LPS group, we found that miR-150 expression was significantly higher in the exosomes and miR-150 agomir groups. Our results showed that exosomes and miR-150 partially attenuated lung inflammation, including total cells, neutrophils, macrophages, TNF-α, IL-6, and IL-1β. H&E staining revealed that exosomes and miR-150 helped maintain the integrity of the alveolar structure. TUNEL staining and Western blotting revealed that exosomes and miR-150 inhibited cell apoptosis in the lung, which was mediated by caspase-3 and Bax/Bcl-2 expression. Exosomes and miR-150 also partially attenuated pulmonary edema, including BALF proteins and the lung wet/dry weight ratio. CD34 is a cell surface sialomucin expressed by vascular, epithelial, and stromal cells. It alleviates hemical- and infection-induced lung damage through maintaining vascular integrity [31]. CD34 and VE-cadherin are closely associated with microvascular endothelial cell injury and changes in lung permeability [32,33]. Immunofluorescence staining for CD34 and VE-cadherin demonstrated that exosomes and miR-150 mitigated microvascular endothelial cell injury. MAPK pathway proteins, including p38, JNK, and Erk, were reportedly activated by LPS in ALI BALB/c mice [34]. In our study, the expression of p-p38, p-JNK, and p-ERK was higher in the LPS group than in the control group, and exosomes and miR-150 suppressed this increase.

In summary, our study showed that MSCs may play a protective role against ALI via the action of exosomes and miRNAs. One of the limitations of our present study is that the number distribution of exosomes was not measured by dynamic light scattering. We demonstrated that miR-150 mitigated LPS-induced ALI by modulating the microvascular endothelial cells and MAPK pathways, indicating that miR-150 could be a potential treatment strategy for ALI.

## Supplementary Material

Supplementary Figure S1

## Data Availability

The datasets used and/or analyzed during the current study are available from the corresponding author on reasonable request.

## Abbreviations

ALI: acute lung injury
Ang: angiopoietin
ARDS: acute respiratory distress syndrome
BALF: bronchoalveolar lavage fluid
BMSC: bone marrow stromal stem cell
H&E: hematoxylin and eosin
IL: interleukin
LPS: lipopolysaccharide
miRNA: microRNA
MSC: mesenchymal stem cell
TNF-α: tumor necrosis factor α
TUNEL: terminal deoxynucleotidyl transferase dUTP nick-end labeling
