# Supplementary material for: Exosomal miR-150 partially attenuated acute lung injury by mediating microvascular endothelial cells and MAPK pathway
Source: Biosci Rep. 2021 Dec 23;42(1):BSR20203363. doi: 10.1042/BSR20203363 (PMC8703023; doi:10.1042/BSR20203363)
Supplement: Supplementary Figure S1 [file BSR-2020-3363_supp.pdf]

Supplementary material

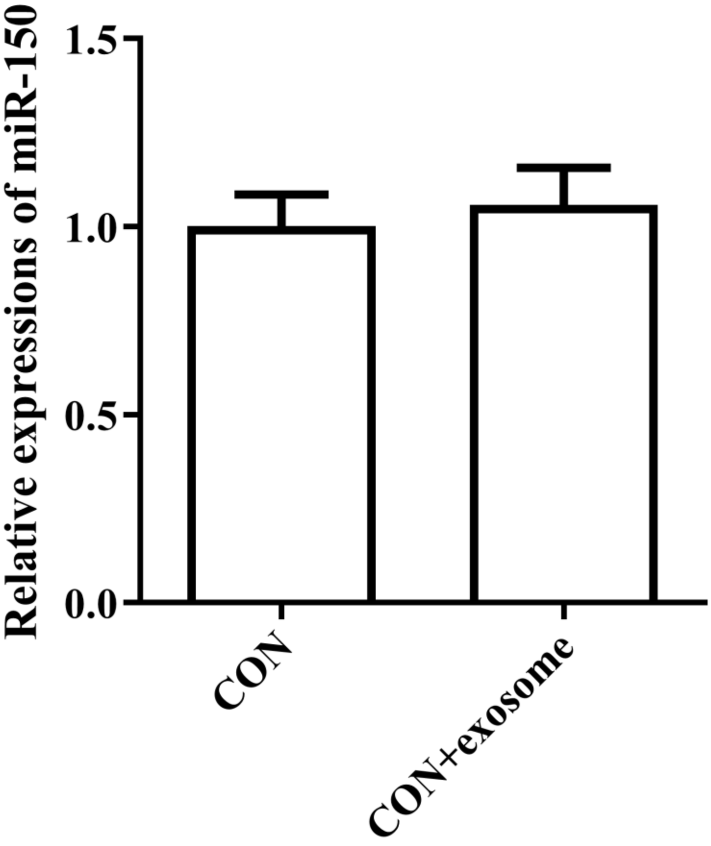

Figure S1. The miR-150 expression in control mice injected with exosomes.

The miR-150 expression in the lungs of mice in the CON, CON + exosome.
